# Nilotinib and Imatinib Are Comparably Effective in Reducing Growth of Human Eosinophil Leukemia Cells in a Newly Established Xenograft Model

**DOI:** 10.1371/journal.pone.0030567

**Published:** 2012-02-14

**Authors:** Daniel Wicklein, Nuno Ramos Leal, Johannes Salamon, Mohammed Thamer, Harald Herrmann, Peter Valent, Udo Schumacher, Sebastian Ullrich

**Affiliations:** 1 Institute of Anatomy and Experimental Morphology, University Cancer Center, University Medical-Center Hamburg-Eppendorf, Hamburg, Germany; 2 Institute of Clinical Chemistry, University Medical-Center Hamburg-Eppendorf, Hamburg, Germany; 3 Department of Radiology, University Medical-Center Hamburg-Eppendorf, Hamburg, Germany; 4 Ludwig-Boltzmann Cluster Oncology, Vienna, Austria; 5 Division of Hematology and Hemostaseology, Department of Internal Medicine I, Medical University of Vienna, Vienna, Austria; Emory University, United States of America

## Abstract

We developed a xenograft model of human Chronic Eosinophilic Leukemia (CEL) to study disease progression and remission-induction under therapy with tyrosine kinase inhibitors using imatinib and nilotinib as examples. The FIP1L1/PDGFRA+ human CEL cell lineEOL-1 was injected intravenously into scid mice, and MR imaging and FACS analysis of mouse blood samples were performed to monitor disease development and the effects of imatinib and nilotinib. Organ infiltration was analyzed in detail by immunohistochemistry after sacrifice. All animals developed CEL and within one week of therapy, complete remissions were seen with both imatinib and nilotinib, resulting in reduced total tumor volumes by MR-imaging and almost complete disappearance of EOL-1 cells in the peripheral blood and in tissues. The new model system is feasible for the evaluation of new tyrosine kinase inhibitors and our data suggest that nilotinib may be a valuable additional targeted drug active in patients with FIP1L1/PDGFRA+ CEL.

## Introduction

Chronic Eosinophilic Leukemia (CEL) is the most frequent variant of myeloproliferative hypereosinophilic syndrome [1], [2]. Neoplastic eosinophils in CEL display PDGFRA fusion genes in most cases [3], [4], [5], [6]. Most common are FIP1L1-PDGFRA (F/P) fusions on 4q12, resulting in F/P^+^ leukaemia [3], [5], [6]. The FIP1L1-PDGFRA fusion protein is considered to cause both a constantly upregulated cell proliferation and an increased survival due to resistance to apoptosis of neoplastic eosinophils [7], [8].

For treatment of CEL, the tyrosine kinase inhibitor imatinib can be used as first line therapy leading to rapid remission of eosinophilia in most cases, and thus reducing organ infiltration with the eosinophilic leukocytes [1], [3], [9].

EOL-1, a cell line established from the peripheral blood of a patient suffering from CEL [8], has been used as an *in vitro* model for the study of F/P^+^ CEL [7]. In vivo EOL-1 cells form palpable tumors after subcutaneous injection in severe combined immunodeficient (SCID) mice, and growth of these tumors can be inhibited by tyrosine kinase inhibitors [10], [11]. EOL-1 cells also show hematologic engraftment after intravenous injection. The latter, however, has only been demonstrated in irradiated NOD/SCID mice so far [12].

As CEL is rare [1], it is rather difficult to recruit the number of patients needed for studies to compare the effectiveness of different (tyrosine kinase) inhibitors in treatment of the disease. Therefore, aim of this study was the development of a human CEL xenograft in immunodeficient mice without the need for irradiation in order to study disease progression and remission under therapy with tyrosine kinase inhibitors. For this purpose we used scid mice, which have previously been more effective in treatment studies than NOD/SCID mice [13]. We also show that nilotinib and imatinib are comparably effective in this animal xenograft model. Imatinib and nilotinib are inhibitors of the typrosine kinase activity of PDGFR, KIT and ABL/BCR-ABL, but with a different selectivity profiles [14]. Both are registered for the treatment of chronic myeloid leukemia.

## Results

### Nilotinib is comparably effective against EOL-1 cells *in vitro* as imatinib

**Figure 1:**
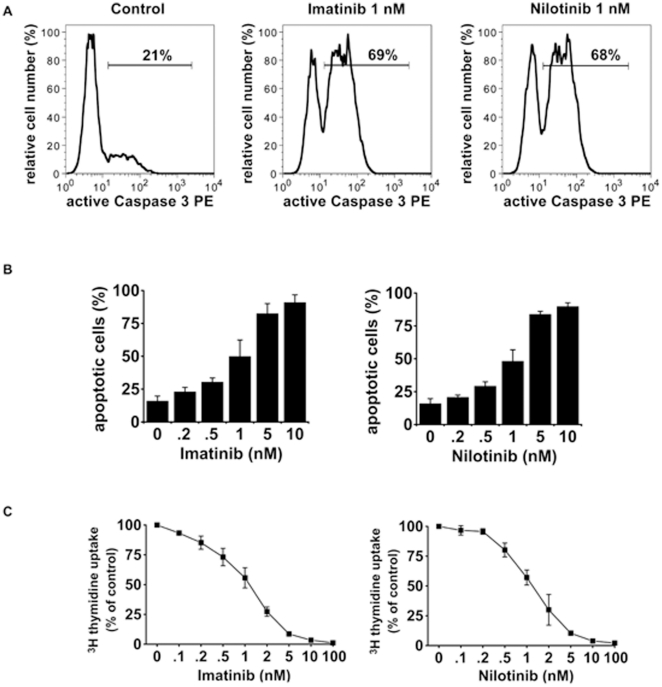
Induction of apoptosis and inhibition of proliferation of EOL-1 cells by imatinib and nilotinib *in vitro*. (A) EOL-1 cells were cultured in medium supplemented with 1 nM imatinib (middle panel) or 1 nM nilotinib (right panel) for 48 hours. Fractions of active caspase 3 positive cells were quantified by flow cytometry. One typical experiment from three independent experiments is shown. (B) EOL-1 cells were cultured in the absence (0) or presence of various concentrations of imatinib (left panel) or nilotinib (right panel). After incubation, cells were examined for the percentage of apoptotic cells by light microscopy. Results represent the mean±S.D. from three independent experiments. (C) Dose-dependent effects of imatinib (left panel) and nilotinib (right panel) on proliferation of EOL-1 cells. Cells were kept in control medium (0) or various concentrations of imatinib or nilotinib for 48 hours. Thereafter, ^3^H-thymidine uptake was measured. Results show the percent 3H-thymidine uptake in drug-exposed cells relative to control (100%) and represent the mean±S.D. from 3 independent experiments.

Imatinib and nilotinib both effectively induced apoptosis in the human CEL cell line EOL-1 *in vitro* (Figure 1A). The apoptosis inducing effects of both drugs were found to be dose-dependent and of comparable magnitude (Figure 1B). Both drugs were also found to inhibit proliferation of EOL-1 cells with almost identical IC50 values (Figure 1C).

### Establishing the CEL model

All mice receiving injections of EOL-1 cells developed systemic CEL. In the therapy group with a total of 11 animals in parts A and B of the experiment four animals died before the end of the experiment on day 35. Of these, one mouse died during anesthesia after MR imaging and three died overnight after the start of therapy on day 27. These mice all showed massive chloroma in the MR images.

In the placebo group, five of 11 mice did not reach the end of the experiment. Of these, one died overnight after the start of treatment on day 28 and four had to be euthanized at some point between days 28 and 35 due to reaching the endpoint criteria as stated below. Upon necropsy, all of the latter animals showed massive chloroma and EOL-1 infiltrations in various organs (see below).

### Analyzing imatinib versus placebo therapy

#### Flow cytometry of blood samples

**Figure 2:**
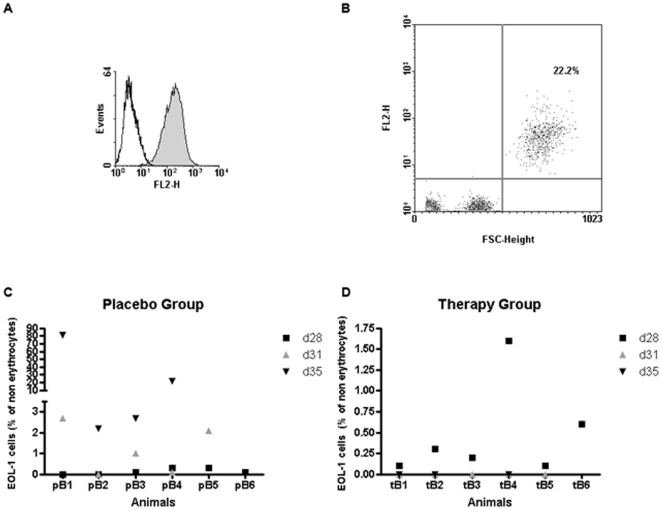
Flow Cytometry of blood samples. (A) EOL-1 cells grown in cell culture express HLA-DR: Histogram for anti-HLA-DR staining (filled curve) versus isotype control (open curve). (B) EOL-1 cells could also be detected in mouse blood after xenotransplantation of EOL-1 cells. Staining of EOL-1 cells with anti HLA-DR from the blood of mouse pB4 on day 35 after erythrolysis is shown. Human EOL-1 cells can clearly be distinguished from murine leukocytes by size (forward scatter, FSC) and HLA-DR positivity (FL2-H). EOL-1 cells (upper right quadrant) constitute 22.2% of non-erythrocyte cells in mouse blood. (C, D) Percentage of EOL-1 cells in mouse leukocytes (part B of the experiment) under imatinib treatment for the placebo group (C) and the therapy group (D). Values are shown for day 28 (d28, black square), day 31 (d31, grey triangle) and day 35 (d35, black triangle). Missing value for a mouse on a given day indicates that the mouse was no longer alive at this point of time.

Human HLA-DR was chosen as a marker for detecting EOL-1 cells in blood and tissue samples of imatinib or placebo treated mice, in order to avoid cross reactions with murine cells. EOL-1 cells from cell culture clearly displayed HLA-DR expression on their surface as analyzed by flow cytometry (Figure 2A). EOL-1 cells could also be detected in blood samples of the animals by staining with anti-HLA-DR. Flow cytometric analysis of a blood sample from mouse pB4 on day 35 is shown as an example (Figure 2B).

The percentage of EOL-1 of the non-erythrocyte cells in the mouse blood was analyzed by flow cytometry. On day 28 after injection, EOL-1 cells ranging from below 0.1 to 4% of the non-erythrocytes could be detected in all animals. For part B of the experiment, this percentage dropped to zero in the blood of the imatinib-treated mice already on day 31 (4 days after therapy start) and EOL-1 cells were still undetectable on day 35 (therapy day 8) in the therapy group (Figure 2C). In the placebo treated group, the percentage of EOL-1 cells increased from days 28 to 31 and even more so to day 35, their percentage ranging from 2.2 to 81% (Figure 2D). On day 35, two animals (pB1 and pB4) appeared apathetic and in generally poor condition, which was due to a manifest blast crisis (81% and 22% EOL-1 cells in the animals' blood, Figure 2B, C); these animals had to be sacrificed according to the animal welfare guidelines.

#### MRI

**Figure 3:**
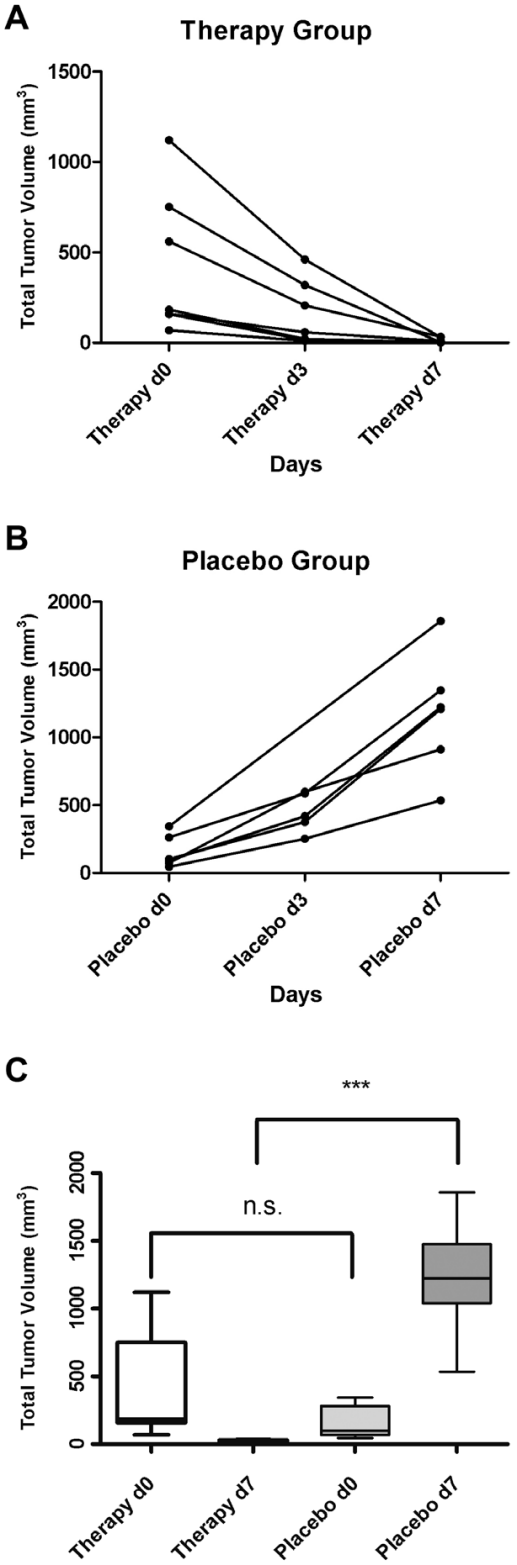
Development of total chloroma volume as determined by MR imaging under imatinib treatment. (A) Total chloroma volume in mm^3^ for each animal from the therapy group on days 27 (Therapy d0), 30 (Therapy d3) and 34 (Therapy d7). (B) Total chloroma volume in mm^3^ for each animal from the placebo group on days 27 (Placebo d0), 30 (Placebo d3) and 34 (Placebo d7). (C) Total chloroma volume in mm^3^ for the therapy and placebo group before (d0) and after (d7) imatinib treatment. n.s.: non-significant, P = 0.1301; ***: highly significant, P<0.0001.

On day 27 (therapy day 0), massive tumor masses could be detected in all mice by MR imaging. Under imatinib therapy the total tumor volume (given in mm^3^) decreased from a mean of 428.9±149.1 on day 27 (day 0 after start of therapy; range 1120.1 to 69.0 mm^3^) to 156.0±67.4 on day 30 (therapy day 3; range 461.0 to 10.3 mm^3^) and further to 11.9±5.2 on day 34 (therapy day 7; range 0.6 to 33.1 mm^3^, Figure 3A).

**Figure 4:**
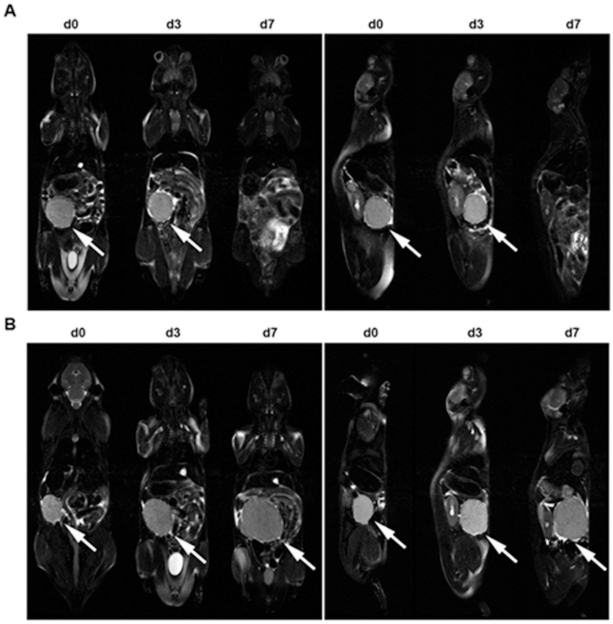
MR imaging. MR images of one mouse each of the therapy (A, imatinib) and placebo group (B). Coronar (left panels) and sagittal (left panels) T2 images of EOL-1 bearing scid mice on days 27 (d0 of treatment), 30 (d3 of treatment) and 34 (d7 of treatment). In the selected plane, remission (therapy) or growth (placebo) of a large abdominal chloroma can be observed (arrows). Chloroma is in complete remission on day 34 under imatinib treatment (A, d7).

In contrast, the tumor volumes drastically increased in the placebo-treated group (again given in mm^3^): From 153.6±49.1 on day 27 (range 45.1 to 343.9 mm3) to a mean of 446.8±65.7 on day 30 (range 252.6 to 599.1 mm^3^) and to a mean of 1233±172.4 on day 34 (range 536.1 to 1858.8 mm^3^; Figure 3B). The differences in mean total tumor volume between the therapy and the placebo group were highly significant both on day 30 (therapy day 3) and 34 (therapy day 7; p<0.0001). Mean total tumor volumes before and after 7 days of therapy for both groups are given in Figure 3C. MR imaging on therapy days 0, 3 and 7 is shown for one typical animal of each group in Figure 4.

#### Histology

**Figure 5:**
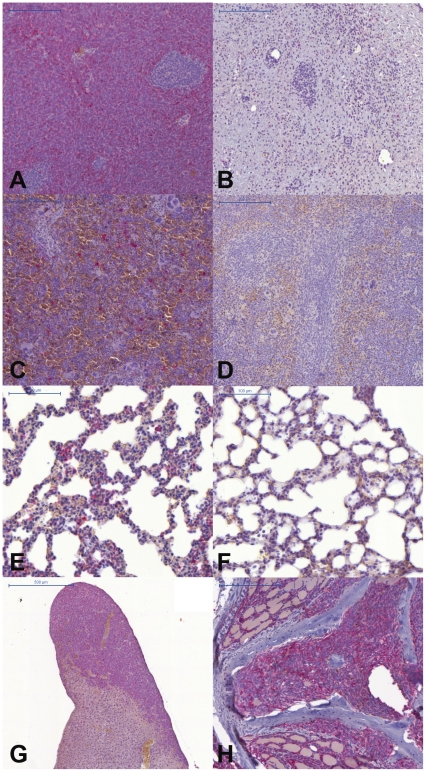
Histology. The presence of human EOL-1 cells was ascertained by labeling for human HLA-DR (seen in red) in tissue-sections. (A) chloroma, placebo group; (B) former chloroma, therapy group with no viable EOL-1 cells detectable; (C) spleen, placebo; (D) spleen, therapy, with no detectable EOL-1 cells; (E) lung, placebo; (E) lung, therapy, with no detectable EOL-1 cells; (F) liver, placebo, massive EOL-1 infiltrates; (G) spine, placebo, with EOL-1 infiltrates in bone marrow.

On day 35 (therapy day 8) all animals were sacrificed. Staining for human HLA-DR revealed EOL-1 cells in sections of paraffin-embedded tissue samples. Chloromas (granulocytic sarcomas) constituted large proportions of the total tumor volume in most animals, however, in the imatinib-treated mice most of the chloromas had gone into complete remission after seven days of therapy. In chloromas from mice of the placebo group, most of the cells were EOL-1 (HLA-DR positive, Figure 5A) with only few mouse cells remaining, whereas almost no EOL-1 cells could be detected in sections of the few remains of chloromas from the therapy group (Figure 5B). EOL-1 cells could be detected in spleen and lung of the placebo-treated mice (Figure 5C, E), but not in the imatinib-treated animals (Figure 5 D, F). Massive infiltrations of EOL-1 cells were also found in liver (Figure 5G) of all mice and in the spine of some (Figure 5H). The latter animals developed paraplegia, which was reversible when the animals were treated with imatinib.

### Using the newly established xenograft model for the evaluation of Nilotinib-treatment of CEL

**Figure 6:**
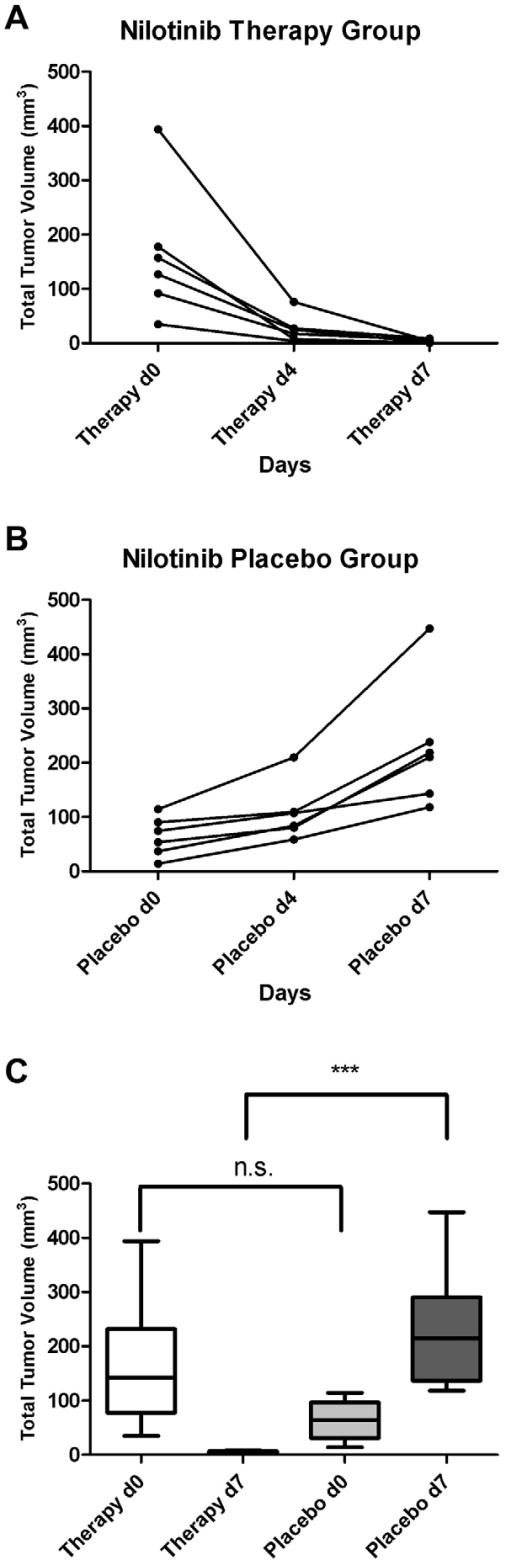
Development of total tumour volume as determined by MR imaging under nilotinib treatment. (A) Total tumour volume in mm^3^ for each animal from the therapy group on days 27 (Therapy d0), 31 (Therapy d4) and 34 (Therapy d7). (B) Total tumour volume in mm^3^ for each animal from the placebo group on days 27 (Placebo d0), 31 (Placebo d4) and 34 (Placebo d7). (C) Total tumour volume in mm^3^ for the therapy and placebo group before (d0) and after (d7) nilotinib-treatment. n.s.: non-significant, P = 0.0869; ***: highly significant, P = 0.0008, unpaired t-test.

For the evaluation of nilotinib for CEL-therapy, a total of six mice per group were orally treated with either nilotinib or placebo, respectively. Analysis of blood samples and MR imaging were performed as described above. EOL-1 cells were not detectable in mouse blood after 4 days of nilotinib treatment, whereas low percentages of EOL-1 cells (0.1 to 1.2%) were detectable in the placebo group. Total tumor volume as determined by MR images was drastically reduced after 7 days of therapy in all nilotinib treated mice. Development of total tumor volume for each animal are shown Figure 6A and in Figure 6B for the placebo group.

Given in mm^3^ the mean total tumor volumes for the nilotinib treated group from days 27, 31 and 34 (therapy days 0, 4 and 7) were 163.8±50.4, 26.2±10.7 and 3.4±1.4 and 63.93±14.89, 108.3±24.2 and 229.3±47.6 for the placebo group, respectively. Both on day 30 (therapy day 4) and 34 (therapy day 7) the differences between the therapy and the placebo group were highly significant (P<0.01, unpaired t-test, Figure 6C). Again some animals showed signs of paraplegia, which were reversible under nilotinib treatment.

## Discussion

Clonal proliferation of eosinophilic leukocytes related to the Fip 1 alpha/BCR ABL mutation represents the most common form of CEL, representing the myeloproliferative subtype of the hypereosinophilic syndrome (HES) [6]. Epidemiologic data demonstrate CEL to be a very rare disorder [1], [3], [5], [6] and since testing for these mutations in HES has not been performed frequently, incidence rates are difficult to estimate. The low incidence and the absence of screening for mutations both contribute to the fact that large cohort studies to evaluate new drug therapies in HES are lacking. Therefore the development of an animal model, closely resembling human disease is essential to evaluate new therapeutic strategies *in vivo*. To our knowledge so far only the NOD/SCID mouse model demonstrated that immunodeficient mice are capable to host EOL-1 cells in the murine circulation [12]. While this model allowed insights into the proliferation and differentiation of EOL-1 cells in the mouse circulation, its practical value is clearly limited as a lethal irradiation of the mice prior to the EOL-1 transfer had to be performed. We therefore switched to SCID mice, which were more suited to therapeutical intervention than NOD/SCID mice in a human T-cell leukemia xenograft [13].

New in our model is the monitoring of the therapy in the mouse blood using FACS analysis, which was not performed in previous models using tyrosine kinase inhibitors or aurora inhibitors, respectively [11], [15].

Our *in vitro* data (Figure 1) showed comparable potency of imatinib and nilotinib against EOL-1 cells, which is in line with data from literature for other cell lines [14]. In human patients, treatment of CEL with imatinib is well established [7], [9], however, resistance of EOL cells to imatinib obviously does occur in an increasing number of cases [16], [17]. First case reports show an efficacy of nilotinib in these cases [18]. In addition, the growth of the EOL -1 cells/tumors at various sites could be monitored by MR imaging for the first time and these findings could be verified by histology.

In patients suffering from CEL, relevant clinical symptoms and organ involvement vary, with splenomegaly being the only common finding [19]. Although we could show the presence of EOL-1 cells in the spleen of all mice from the placebo group (Figure 4), we did not observe pronounced splenomegaly in the animals. However, it has to be kept in mind that the role of the murine spleen in hematopoesis differs from that in humans [20]. In FIP1L1/PDGFRA+ CEL, endomyocardial infiltrates of CEL cells are often observed. However, we could not find EOL-1 cells in sections of the animals' hearts. EOL-1 cells were always detectable in liver and spleen of the animals from the placebo group and chloromas at various locations were always detected in MR imaging of the mice. Chloromas (granulocytic sarcomas) have been described in human CEL [21] and their incidence might be underestimated in AML and generally in CML [22] as the majority of chloromas is only diagnosed at autopsy [23]. We could show that remission of EOL-1 leukemia was achieved by only a one week treatment with intraperitoneal imatinib or with oral nilotinib treatment in our xenograft model, respectively. With both tyrosine kinase inhibitors, the mice showed no signs of disease anymore (even paraplegia – if any – vanished after treatment). Both drugs were comparably efficient in eliminating CEL cells from the animals' blood, organs and from the few remnants of chloroma that could still be found at the necropsies. It is interesting that both tyrosine kinase inhibitors showed a high efficacy in the treatment of these solid nodules. The particular value of the new model lies in the fact that the course of disease can be easily monitored using both FACS and MR imaging.

Many studies have demonstrated tyrosine kinase inhibitors to be highly effective in treatment of CEL (Valent review, das polnische Paper). Long term remissions with molecular remissions can be achieved in more than 90% [1], [19]. However, after successful induction of remission it still remains unclear how long drug application has to be continued as a maintenance therapy [1], [19], [24], [25]. Despite the great benefit of tyrosine kinase inhibitors, many studies report small numbers of patients who develop resistance to tyrosine kinase inhibitor treatment which is associated with a poor prognosis [17], [19]. The mouse model presented in this work will allow short term experiments to investigate treatments with new (tyrosine kinase) inhibitors as well as long term studies to understand the induction and maintenance of remission and the development of resistance towards tyrosine kinase inhibitors.

## Materials and Methods

### Tyrosine Kinase Inhibitors and other Reagents

The tyrosine kinase inhibitors imatinib (Gleevec®) and nilotinib (Tasigna®) were kindly provided by Novartis (Basel, Switzerland). RPMI 1640 medium and fetal calf serum (FCS) were purchased from PAA laboratories (Pasching, Austria), ^3^H-thymidine from GE Healthcare (Buckinghamshire, UK), and an antibody against activate caspase 3 (C92-605) from Becton Dickinson Biosciences (San Jose, CA, USA).

### Cell Culture

EOL-1 cells (DSMZ, Braunschweig, Germany) were cultured in RPMI 1640 medium, supplemented with 10% FCS, 2 mM L-glutamine, 100 Uml^−1^ penicillin and 100 µgml^−1^ streptomycin (all Invitrogen, Karlsruhe, Germany) at 37°C in a humidified atmosphere of 5% CO_2_. To investigate drug effects, EOL- 1 cells were incubated with control medium or in various concentrations of imatinib (0.1–100 nM) or nilotinib (0.1–100 nM) at 37°C for 48 hours. Thereafter, the number of apoptotic cells and ^3^H-thymidine uptake were measured. All experiments were performed at least three times. In select experiments, expression of active caspase 3 in EOL-1 cells was determined by flow cytometry after drug exposure.

### Animals

All animals used were pathogen-free Balb/c severe combined immunodeficient scid mice aged 9–14 weeks with a weight of 25–30 g at the beginning of the experiments. The animals were housed in filter-top cages and provided food and water ad libitum.

### Ethics Statement

The methodology for carrying out the animal experiments was consistent with the UKCCR guidelines for the welfare of animals in experimental neoplasia [26]. The experiment was supervised by the institutional animal welfare officer, and approved by the local licensing authority (Behörde für Soziales, Familie, Gesundheit, Verbraucherschutz; Amt für Gesundheit und Verbraucherschutz; Billstr. 80, D-20539 Hamburg, Germany) under the project no. 41/08.

### Establishing a mouse model for the treatment of human CEL with Imatinib

The experiment (**part A**) was first carried out with 10 mice (5 animals for the therapy and the placebo group, respectively) and repeated (**part B**) with 12 mice (6 animals each group). The mice were designated with the letter **t** (for therapy group) or **p** (placebo group) and a running number. In the following, each individual animal will be referred to as for example “tB3” (therapy group, experiment part B, mouse number 3). For injection, EOL-1 cells were washed and resuspended in PBS at 2×10^7^ cells per ml. All mice received an intravenous injection of 2×10^6^ EOL-1 cells (in 100 µl PBS) and were either treated with intraperitoneal injections of imatinib (100 mg/kg body weight in 100 µl PBS) or PBS as placebo control. The animals each received one injection per day for 10 days.

27 days after the injection of the EOL-1 cells, the first MR imaging was acquired and therapy started on the next day. MR imaging was repeated on days 30 and 34. Additionally, blood was taken on day 28 (experiment part A) and additionally on days 31 and 35 (part B only). All mice were sacrificed on day 35 after 7 days of treatment.

### Nilotinib treatment

The animal experiment using nilotinib for EOL-treatment was performed as described for imatinib above, except as follows.

Nilotinib was applied orally via gavage once per day for 7 days (100 mg/kg body weight in 100 µl sterile water), placebo control was sterile water.

### MR imaging

The animals were anesthesized for approximately 45 minutes by intraperitoneal injection of a weight-adapted dose (1 µl/g body-weight) of a mixture of 1.2 ml Ketamin (Gräub AG., Bern, Manley CH), 0.8 ml Rompun (Bayer AG, Leverkusen, Germany) und 8 ml physiological saline solution (Invitrogen). MRI was performed on a clinical 3.0T MRI scanner (Intera, Philips Medical Systems, Best, The Netherlands). The scanner was equipped with a conventional body transmit coil and gradient system allowing a maximal amplitude of 30 mT m^−1^ and a slew rate of 50 mT^−1^ s^−1^. For signal reception a dedicated four-element mouse coil (Philips Research Laboratories, Hamburg, Germany) with an inner diameter of 2.5 cm was used. The MR sequence protocol consisted of a T1 weighted localizer sequence in three orthogonal planes, an additional T2 weighted localizer in sagittal orientation followed by a high-resolution fat saturated T1 weighted 2D turbo spin-echo sequence (TSE) in coronar orientation, a fat saturated T2 weighted 2D TSE in coronar and sagittal orientation. Image parameters of the whole body coronar T1 TSE sequence were as follows: time of repetition (TR)/time of echo (TE)/flip angle (FA) = 1275 ms/33 ms/90°; field-of-view (FOV) = 100×35 mm^2^; matrix = 464×464; slice thickness (ST) = 1 mm, number of slices (NS) = 14; number of acquisitions (NA) = 3; spatial resolution = 0,22×0,22×1 mm; acquisition time = 5 minutes 18 seconds. Image parameters of the fat saturated whole body coronar and sagittal T2 TSE sequence were as follows: TR/TE/FA = 2361 ms/90 ms/90°; FOV = 100×35 mm^2^; matrix = 448×448; ST = 1 mm, NS = 14; NA = 3; spatial resolution = 0,22×0,22×1 mm; acquisition time = 3 minutes 37 seconds. The tumor size was determined by measuring the largest dimensions of the tumors height, length and width in the coronar and sagittal plane.

### Flow Cytometry

Each blood sample for FACS-analysis was prepared from 200 µl of fresh EDTA-blood as follows: Erythrolysis was performed according to the manufacturer's instructions using the Mouse Erythrolysis Kit (R&D, Wiesbaden, Germany). Cells were stained for 30 min on ice with phycoerythrin-labelled mouse anti-human HLA-DR (BD, Heidelberg, Germany), diluted 1∶50 in 100 µl FACS buffer (PBS, 1% BSA) or the corresponding isotype control (Miltenyi, Bergisch-Gladbach, Germany). After washing, cells were subjected to fluorescence assisted flow cytometry on a FACSCalibur (BD). Files were analyzed using Win MDI 2.9 software.

### Immunohistochemistry

Immunohistochemistry using sections of paraffin-imbedded tumours was carried out as previously described [27]. Briefly, after antigen retrieval (microwave, citrate buffer, ph = 6), mouse anti-HLA-DR alpha chain or corresponding isotype control was used as first antibody, respectively, followed by incubation with biotinylated rabbit anti-mouse (all Dako, Glostrup, Denmark) and visualization using the streptavidin-alkaline-phosphatase based Vectastain ABC kit (Vector, Burlingame, CA, USA). Slides were scanned by a Mirax microscope (Zeiss, Jena, Germany) and the Mirax Viewer (Zeiss) software was used to take images.

### Statistical analyses

Statistical Analysis was done using GraphPad Prism 5 (GraphPad, La Jolla, CA, USA). Tests used are given for each value of P in the Results section.
